# Understanding heterogeneity in the host response to *Staphylococcus aureus *infection for prognostic biomarker discovery

**DOI:** 10.1186/cc14017

**Published:** 2014-12-03

**Authors:** JB Dinoso, J Gutierrez, DF Choy, S Kummerfeld, A Baruch, HF Chambers, CM Rosenberger

**Affiliations:** 1 ITGR Diagnostic Discovery, Genentech Inc., South San Francisco, CA, USA; 2 Pharmacodynamic Biomarkers, Genentech Inc., South San Francisco, CA, USA; 3 Bioinformatics, Genentech Inc., South San Francisco, CA, USA; 4 Division of Infectious Disease, University of California San Francisco, San Francisco, CA, USA

## Introduction

Invasive *Staphylococcus aureus *infections remain an unmet medical need with the issues of drug resistance (MRSA) and mortality. Understanding clinical trial data in the development of antibiotics to *S. aureus *is complicated by heterogeneous clinical outcomes (that is, length of hospitalization, mortality, treatment response), which makes interpretation of drug efficacy challenging. Identification of prognostic biomarkers of different biological processes that associate with clinical outcomes would aid in clinical development of novel therapies for *S. aureus *infections.

## Methods

In an effort to discover biomarkers that differentiate patient populations based on clinical outcomes following *S. aureus *infection, we retrospectively analyzed published gene expression datasets of *S. aureus *infection and sepsis.

## Results

We identified a leukocyte gene expression signature that positively correlated with *S. aureus *disease severity [1]. This severity signature was enriched for genes associated with neutrophils but was not solely explained by increased percentage of neutrophils. This set of genes was also associated with severity in sepsis, with higher expression in patients with septic shock compared with sepsis [2] and in nonsurvivors compared with survivors of septic shock [3]. Our *in vitro *studies revealed that the severity signature may reflect an increase in circulating immature neutrophils or band cells which has been previously reported in the context of bacterial stimuli and sepsis [4,5]. This line of evidence is consistent with a recent report by Guerin and colleagues that demonstrated that quantification of immature neutrophils by flow cytometry was prognostic for sepsis mortality [6].

## Conclusion

To extend the insight gained from our retrospective analysis and *in vitro *studies, we are conducting a longitudinal, non-interventional clinical study of patients with *S. aureus *bacteremia. The goal of the study is to associate molecular metrics (gene expression, plasma protein levels, immune cell subsets) with clinical outcomes (length of hospitalization, mortality, treatment response, readmission for recalcitrant infection). Our preliminary data show an association between grade of sepsis or infection localization and increased immature neutrophils as well as monocyte subsets that can promote inflammation or immune exhaustion. Ongoing experiments are designed to understand the impact of these cellular phenotypes on disease progression and to identify robust protein or RNA biomarkers that are prognostic for clinical outcomes.
